# Dietary supplementation with *β*-mannanase and probiotics as a strategy to improve laying hen's welfare

**DOI:** 10.3389/fvets.2022.985947

**Published:** 2022-09-20

**Authors:** Camila Lopes Carvalho, Ines Andretta, Gabriela Miotto Galli, Gabriel Bueno Martins, Nathalia de Oliveira Telesca Camargo, Thais Bastos Stefanello, Raquel Melchior, Marcos Kipper da Silva

**Affiliations:** ^1^Department of Animal Science, Faculdade de Agronomia, Universidade Federal do Rio Grande do sul, Porto Alegre, Brazil; ^2^Elanco Animal Health, São Paulo, Brazil

**Keywords:** additives, animal behavior, image analysis, ethology, poultry

## Abstract

A trend toward animal welfare improvement is observed in animal production, in addition to restrictions imposed on the use of antimicrobials. This study's objective was to evaluate whether β-mannanase and probiotic supplementation can change hen's behavior. Light weight laying hens (36 weeks old) were housed in cages randomly allocated to one of four different treatments: control group, fed non-supplemented diets; diets supplemented with 300 g/ton of β-mannanase; diets supplemented with 50 g/ton of probiotic; or diets containing both 300 g/ton of β-mannanase and 50 g/ton of probiotic. The behavior of 24 birds was recorded for a week using video cameras. The frequency and time of main behaviors (eating, walking, standing, sitting, drinking, and exploring) were analyzed in three periods per day (from 09:00 to 09:15; from 01:00 to 01:15, and from 04:00 to 04:15), as well as the time of other behaviors (leg-stretching and wings, scratching, wing-flapping, aggressive and non-aggressive pecks). Frequency and lesion scores were also analyzed using a visual score of three body regions: neck, tail, and cloaca; as well as comb injuries. β-mannanase was able to increase the frequency of feeding behavior by 49% (*P* < 0.05) and hens also spend 20% (*P* < 0.05) more time in this behavior compared to the control treatment. The use of probiotics also enhanced by 39% (*P* < 0.05) the frequency and 19% the time (*P* < 0.05) and the supplementation with combined additives was able to increase by 29% (*P* < 0.05) the frequency and 25% (*P* < 0.05) the time in feeding behavior. β-mannanase and probiotics also increased the frequency and time spent exploring behavior (*P* < 0.05) and promoted a higher frequency in standing behavior (*P* < 0.05) and decreased the time spent on sitting behaviors (*P* < 0.05). The combined additives showed less frequency and time in sitting behaviors (*P* < 0.05), while increased wing-flapping behavior (*P* < 0.05). All the treatments were able to reduce pecking (*P* < 0.05). Therefore, the addition of β-mannanase and probiotics to laying hen diets is an effective strategy to improve bird welfare.

## Introduction

The poultry industry intensified after the 1940's, generating eggs from caged birds (1). However, it is known that the rearing of birds in cages, mainly due to their reduced space, is not compatible with all the physiological needs of birds and ends up generating a greater susceptibility to stress (2), which has negative effects on the intestinal microbiota balance (3, 4). In broiler chickens, probiotics have already proven to be efficient by reducing heat stress and abnormal behavior and improving their health. Such responses occur from the regulatory power of probiotics under the microbiota-gut-brain axis (5). Thus, the modulation of gut microbiota has become a strategy for improving hosts' health and welfare under various conditions (6). Probiotics also alleviate the stress response along the hypothalamic-pituitary-adrenal axis, reducing plasma or brain levels of corticotropin-releasing hormone, adrenocorticotropic hormone, and corticosterone (4, 7).

Enzyme supplementation is another strategy that can benefit the gut health status by reducing the impacts of anti-nutritional components. The use of β-mannanase can help non-ruminant animals dealing with the non-starch polysaccharides, which can reduce nutrient digestibility (8). Such components are found in plant cell walls and are present in many ingredients largely used in animal feeding Among the main hemicelluloses found in plant cell walls are β-mannans, which can be subdivided into different forms including galactomannan, glucomannan, and glucogalactomannan (9). Soybean meal is probably the best example of an ingredient with high β-mannan levels which is commonly used in the feed industry, being the primary protein source for poultry in most countries. Other ingredients such as palm kernel meal, copra meal, sesame meal, and guar gum/meal also contain high levels of β-mannan, however, these are used in a more regional context compared with soybean meal. Some of those mannans can also be found on the surface of pathogenic microorganisms. Thus, the animal's innate immune system is activated when foods that contain β-mannans are ingested, which responds with the proliferation of monocytes, macrophages, dendritic cells, and increased production of cytokines. Such factors generate an unnecessary energy expenditure and an increase in inflammatory responses (10). By hydrolyzing the β-mannans, this enzyme can improve the digestibility of mannans, increasing the population of beneficial bacteria, improving immunity, digestion and absorption of nutrients, in addition to limiting the proliferation of potential pathogens in the intestine (8).

Improving animal health is one of the most important goals to achieve animal welfare and feed additives can be helpful in this task. Despite the probiotic benefits already described in previous studies, most of the available data was obtained in other poultry categories (i.e., broilers). This is, to our knowledge, the first paper about the influence of β-mannanase on the behavior and welfare of poultry hens. Besides, both additives have complementary action modes, which can indicate the possibility of synergic effects when supplemented together in the feed, and the possible combined effects have not yet been described in the literature. Thus, the aim of this study was to evaluate whether β-mannanase and probiotic supplementation combined or alone can change the behavior and the welfare of commercial laying hens.

## Materials and methods

### Animals, housing and experimental design

This study was conducted at a commercial farm, in Salvador do Sul, state of Rio Grande do Sul, Southern Brazil. The experimental units were randomly selected among the hens housed in a commercial farm with about 28 thousand lightweight laying hens (Hyline W 36 lineage, 36 weeks old). The replicates were assigned in a completely randomized design to the four treatments, that were: control treatment, which consisted of a basal diet, without supplementation with any other additive; β-mannanase, which was the control diet supplemented with 300 g/ton of β-mannanase; probiotic, that was the control diet supplemented with 50 g/ton of a multi-cepa probiotic additive; and β-mannanase + probiotic treatment, which was the control diet supplemented with 300 g/ton of β-mannanase and 50 g/ton of a multi-cepa probiotic additive.

The β-mannanase (Hemicell HT™, Elanco Animal Health, São Paulo, Brazil) consists of an exogenous enzyme from the fermentation of the *Paenibacillus lentus* bacteria. The probiotic additive (Protexin Concentrate™, Elanco Animal Health, São Paulo, Brazil) includes *Lactobacillus acidophilus* (2.06 × 10^8^ UFC/g), *Lactobacillus bulgaricus* (2.06 × 10^8^ UFC/g), *Lactobacillus plantarum* (1.26 × 10^8^ UFC/g), *Lactobacillus rhamnosus* (2.06 × 10^8^ UFC/g), *Bifidobacterium bifidum* (2.0 × 10^8^ UFC/g), *Enterococcus faecium* (6.46 × 10^8^ UFC/g) e *Streptococcus thermophilus* (4.10 × 10^8^ UFC/g).

The basal diet was a corn-soybean meal-based feed formulated according to the nutritional requirements of genetic (11). Inert material (kaolin) was included in the basal feed to replace β-mannanase and/or probiotic additives. Feed and water were both provided *ad libitum* throughout the experimental period using nipple drinkers and gutter feeders.

The birds were housed in conventional sheds, arranged in an east-west direction, with concrete floors and masonry walls complemented with wire mesh to the ceiling. The shed was equipped with side curtains, which were managed according to weather conditions to provide thermal comfort. The average minimum and maximum temperature and air relative humidity values recorded were 18 and 36°C, and 35.8 and 94.7%, respectively. The lighting regime was composed of 16 h of light and 8 h of dark per day.

The birds remained in galvanized-wire cages (100-cm long × 40-cm wide × 45-cm high, resulting in a floor area of 500 cm^2^/hen) throughout the experimental period. Four birds were allocated in each cage. Birds were supplemented for 84 days and the assessments were performed in the last week of the trial.

### Data collection

Behavioral assessments were performed through image capture, combined with local feather scoring and comb abnormalities assessment. The evaluations of behavioral traits were carried out through images captured by four cameras, installed in front of the cages that were analyzed. The image acquisition (recording) process was performed automatically to avoid interfering with the results. The score of feathers and comb abnormalities was performed using the visual score at the end of the trial.

For the behavior evaluation, six birds per treatment (one from each cage) were randomly selected for observation. The captured images were carried out for 7 consecutive days, in a period of 15 min in the morning (09:00 to 09:15); which is related to the highest peak of laying of the birds, plus 30 min in the afternoon, divided into two periods (from 1 pm to 1:15 pm and 4 pm to 4:15 pm) referring to the hottest and cooler times of the day, respectively. Such methodology was adapted from Barbosa Filho et al. (12), Garcia et al. (13), and Pereira et al. (14). The observation of behaviors for 15 continuous minutes was proposed by Bizeray et al. (15).

Images were recorded and stored on media (pen drive) for further analysis by visual counting and frequency method. The images were analyzed by the same observer and, with the aid of a stopwatch, which counted the time of each behavior expressed by the birds. In other words, time spent corresponded to the duration of the various expressions of each behavior category summed per individual. An ethogram (Table 1) adapted from Pereira et al. (14), Rudkin and Stewart (16) was used for the behavioral analyses. The frequency (n) of each behavior was then calculated per individual in each observation interval.

**Table 1:** Behavioral ethogram for laying hens in cage system.

**Activity**	**Behavior**	**Description**	**Measurement**
Stopped	1. Eating	Act of eating continuously	Frequency and time
	2. Walking	Take at least one step in any direction	Frequency and time
	3. Standing	Alert posture or standing in one place	Frequency and time
	4. Sitting	Sitting with the head retracted and eyes open or closed	Frequency and time
	5. Drinking water	Continuous water intake	Frequency and time
Movement	6. Exploring feathers	Exploring feathers with the beak, for maintenance or investigation	Frequency and time
	7. Scratching the head	Behavior in which the bird scratches its head with one of its paws	Frequency
	8. Wing-flapping	Beating, stretching, shaking, and ruffling the feathers	Frequency
	9. Leg-stretching	Movement of stretching one leg and one wing, from the same hemisphere of the body	Frequency
	10. Stretching	Act of stretching one of the wings or legs	Frequency
	11. Non-aggressive peck	Light pecks aimed at other birds, usually in the head region or other parts of the body	Frequency
	12. Aggressive peck	Strong pecks from another bird cause tissue damage to the birds and/or damage to their combs	Frequency

The lesion score was performed through a visual score attributed to three body regions: neck, tail, and vent from 25 birds per treatment (randomly selected). Possible injuries and different degrees of severity were analyzed using a scale from 0 to 5, with the best score being 0 (complete plumage, no damage) and the worst score being 5 (completely feathered areas with skin lesions). The methodology was adapted from Dennis et al. (17) and Larsen et al. (18). Comb abnormalities were observed in the same birds using the method proposed by (19–21) Ali and Cheng (19), Struthers (20), and Welfare Quality (21), which was adapted. In this test, the same 25 birds per treatment were analyzed using a scale from 0 to 3, with the best score being 0 (no evidence of comb abnormalities) and the worst score being 3 or more comb areas with evidence of abnormalities).

All behavioral tests were carried out in the last week of the experiment, allowing the birds to remain exposed to the treatments for a longer period. The same animal was used only in one of the tests, thus preventing one test from interfering with the result of the other.

### Statistical analyses

Data analyzes were performed using the SAS statistical program. Behavior data were submitted to variance analyses using PROC GLIMMIX considering the effects of treatment, time of day, and their interaction. Behavior data were collected in the same animal for 7 days, their analysis was performed considering repeated measures over time. The PROC GLIMMIX was also used to evaluate the feather and comb abnormality scores. For these responses, only the treatment effect was considered. All residuals were tested for normality. Eventual differences were compared by Tukey test at 1 and 5% levels. Tendencies were also highlighted in the text considering a 10% level.

## Results

Animals performed according to the expected for their genotype throughout the entire trial (data not shown). During the experimental period, no severe health problems were observed.

The layer's behavior was evaluated in this trial at three different times of the day. The animals showed higher eating (*P* < 0.05) and drinking (*P* < 0.01) frequencies and spent more time in these behaviors (*P* < 0.05 for eating; *P* < 0.01 for drinking) at 1:00 pm. Lower frequencies of standing (*P* < 0.01) and exploring (*P* < 0.01) behaviors were found at 4:00 pm, while birds showed a higher frequency of sitting (*P* < 0.01) at the same time of the day. Walking, scratching, wing-flapping, leg-stretching, wing stretching, as well as aggressive and non-aggressive peck behaviors were similar throughout the day.

### Effect of feeding additives on the main behaviors

Birds supplemented with β-mannanase increased (*P* < 0.01) the frequency of eating behavior by 49% compared to the control group, while probiotics enhanced this response by 39%, and combined additives by 29% (Table 2). The time spent on eating behavior was increased (*P* < 0.01) by β-mannanase in 20%, by probiotics in 19%, and by combined additives in 25% (Table 3). An interaction ‘time vs. time of observation' (*P* < 0.01) was observed for eating frequency and time spent in this behavior. In this particular, the treatments were not able to modify the eating frequency during the laying pick (09:00 am), while no treatment effect was found in the time expended eating during the hottest time of the day (1:00 pm).

**Table 2:** Frequency of the main behaviours^1^ observed in laying hens fed β-mannanase (βM) and/or probiotics (PB)^2^.

**Traits**	**Treatments**				***Avg time***	***P*** **-value** ^**3**^		
	**Control**	**βM**	**PB**	**βM+PB**		**Treat**	**Obs**	**T × O**
**Eating**								
9 h^4^	2.07	2.39	2.40	2.50	2.34^X^	<0.001	0.034	0.001
13 h^4^	1.86^C^	4.26^A^	2.88^B^	2.34^B^	2.83^Y^	SE^5^=0.16
16 h^4^	1.07^B^	3.18^A^	2.98^A^	2.25^A^	2.37^X^			
*Avg treat*	1.67^B^	3.28^A^	2.75^A^	2.36^A^				
**Walking**								
9 h	0.90^B^	0.33^B^	1.14^A^	0.62^B^	0.75	<0.001	0.282	0.043
13 h	0.88^A^	0.37^B^	0.93^A^	0.42^AB^	0.64	SE=0.12
16 h	0.60	0.55	0.81	0.40	0.58			
*Avg treat*	0.80^A^	0.42^B^	0.96^A^	0.48^B^				
**Standing**								
9 h	1.71^B^	2.61^A^	2.83^A^	2.19^AB^	2.33^X^	<0.001	0.003	0.071
13 h	1.64^B^	1.98^AB^	2.48^A^	2.05^AB^	2.04^XY^	SE=0.17
16 h	0.93^B^	1.81^A^	2.07^A^	2.40^A^	1.80^Y^			
*Avg treat*	1.43^B^	2.13^A^	2.46^A^	2.21^A^				
**Sitting**								
9 h	0.48	0.48	0.50	0.16	0.40^Y^	0.012	0.001	0.035
13 h	0.59	0.26	0.59	0.55	0.50^Y^	SE=0.10
16 h	1.19^A^	0.49^B^	0.62^B^	0.75^AB^	0.76^X^			
*Avg treat*	0.75^A^	0.41^B^	0.57^AB^	0.49^B^				
**Drinking**								
9 h	0.95	0.89	0.81	1.13	0.95^Y^	<0.001	0.003	0.009
13 h	1.05^B^	2.35^A^	1.67^AB^	1.06^B^	1.53^X^	SE=0.14
16 h	0.21^B^	1.54^A^	1.12^A^	1.56^A^	1.01^Y^			
*Avg treat*	0.74^B^	1.59^A^	1.20^AB^	1.11^AB^				
**Exploring**								
9 h	0.57^BC^	1.25^A^	0.95^AB^	0.35^C^	0.78^X^	<0.001	0.002	0.040
13 h	0.28^B^	0.99^A^	0.55^B^	0.49^B^	0.58^XY^	SE=0.09
16 h	0.07	0.44	0.43	0.55	0.37^Y^			
*Avg treat*	0.31^B^	0.89^A^	0.64^A^	0.46^AB^				

^1^Times that each bird performed the behavior during the observation window (15 min).

^2^Means followed by different uppercase letters differ statistically at 5%, while lowercase letters were used to indicate differences at 10%. Comparisons were performed among treatments – lines^A, B, C, D^ within each observation time and also for averages obtained when the three observation times were polled together (indicated as ‘Avg treat'). The averages obtained when the four treatments were polled together in each observation time are also presented (indicated as ‘Avg time') and compared within the column^X, Y, Z^.

^3^Probability of treatment effect (treat), time of observation (obs), and interaction (T × O).

^4^9 h refers to the observation period from 9 AM to 9:15 AM, 13 h refers to the observation period from 13 PM to 13:15 PM, while 16 h refers to the observation period from 16 PM to 16:15 PM.

^5^Standard error.

**Table 3:** Time expended (minutes/bird)^1^ in each of the main behaviors by laying hens fed β-mannanase and/or probiotics^2^.

**Traits**	**Treatments**				***Avg time***	***P*** **-value** ^**3**^	
	**Control**	**βM**	**PB**	**βM+PB**		**Treat**	**Obs**	**T × O**
**Eating**								
9 h^4^	7.51^B^	5.91^B^	6.99^B^	9.81^A^	7.56^XY^	0.004	0.016	<0.001
13 h^4^	7.39	8.47	7.94	9.25	8.26^X^	SE^5^=0.42
16 h^4^	3.85^B^	8.98^A^	8.24^A^	5.87^AB^	6.73^Y^			
*Avg treat*	6.25^B^	7.79^A^	7.72^A^	8.31^A^				
**Walking**								
9 h	0.84^a^	0.37^b^	0.88^a^	0.43^ab^	0.63	<0.001	0.330	0.065
13 h	1.05^A^	0.50^AB^	0.89^A^	0.25^B^	0.67	SE=0.12
16 h	0.78^a^	0.49^ab^	0.53^ab^	0.25^b^	0.51			
*Avg treat*	0.90^A^	0.45^BC^	0.77^AB^	0.31^C^				
**Standing**								
9 h	3.26^AB^	4.78^A^	4.13^A^	2.52^B^	3.67^X^	0.259	0.028	<0.001
13 h	3.03	2.77	2.94	2.38	2.78^Y^	SE=0.38
16 h	2.25^B^	2.19^B^	3.64^AB^	4.15^A^	3.06^XY^			
*Avg treat*	2.85	3.25	3.57	3.02				
**Sitting**								
9 h	1.07	1.99	0.89	0.62	1.14^Y^	<0.001	<0.001	<0.001
13 h	2.02	0.56	0.68	0.86	1.03^Y^	SE=0.24
16 h	7.82^A^	1.50^BC^	0.65^C^	2.77^B^	3.19^X^			
*Avg treat*	3.64^A^	1.35^B^	0.74^B^	1.42^B^				
**Drinking**								
9 h	1.49^A^	0.46^B^	0.53^B^	1.34^A^	0.96^Y^	0.352	0.002	<0.001
13 h	1.24	1.79	1.75	1.48	1.56^X^	SE=0.14
16 h	0.25^B^	1.31^A^	1.24^A^	1.27^A^	1.02^Y^			
*Avg treat*	0.99	1.19	1.17	1.36				
**Exploring**								
9 h	0.70^BC^	1.46^AB^	1.69^A^	0.29^C^	1.04^X^	0.008	0.005	0.012
13 h	0.28	0.91	0.53	0.81	0.64^XY^	SE=0.14
16 h	0.07	0.36	0.67	0.63	0.43^Y^			
*Avg treat*	0.35^B^	0.91^A^	0.96^A^	0.58^AB^				

^1^Times that each bird performed the behavior during the observation window (15 min).

^2^Means followed by different uppercase letters differ statistically at 5%, while lowercase letters were used to indicate differences at 10%. Comparisons were performed among treatments – lines^A, B, C, D^ within each observation time and also for averages obtained when the three observation times were polled together (indicated as ‘Avg treat'). The averages obtained when the four treatments were polled together in each observation time are also presented (indicated as ‘Avg time') and compared within the column^X, Y, Z^.

^3^Probability of treatment effect (treat), time of observation (obs), and interaction (T × O).

^4^9 h refers to the observation period from 9 AM to 9:15 AM, 13 h refers to the observation period from 13 PM to 13:15 PM, while 16 h refers to the observation period from 16 PM to 16:15 PM.

^5^Standard error.

The walking behavior (frequency and time) of birds supplemented with probiotics was similar to the control treatment. However, birds fed diets containing β-mannanase and combined additives presented lower (*P* < 0.01) frequency and spent less time in this behavior. The ‘time vs. time of observation' interaction was found for the walking frequency (*P* < 0.05) and tended to happen also for the time spent walking (*P* < 0.10), indicating that treatment effects are not constant throughout the day.

All supplemented treatments increased the frequency of the standing behavior (*P* < 0.01). β-mannanase increased it by 49%, probiotics by 72%, and combined additives by 54%. The time spent standing was not affected by the treatments. Interaction ‘time vs. time of observation' was observed for the time spent standing (*P* < 0.001) and a tendency was also found for the behavior frequency.

Supplemented treatments showed a lower frequency of sitting behavior (*P* < 0.05), with β-mannanase decreasing this response by 45% and combined additives by 35%. The time spent in sitting behavior was also decreased (*P* < 0.01) by β-mannanase (-62%), probiotics (-80%), and combined additives (-60%). Interactions ‘time vs. time of observation' were found for both responses (*P* < 0.05 for frequency; *P* < 0.01 for time), with treatment effects occurring at 4:00 pm.

Birds supplemented with β-mannanase increased (*P* < 0.01) the frequency of drinking behavior by 53% compared to the control group, while other supplemented treatments showed intermediary results. Treatments were not able to modify the overall time spent drinking. However, interactions ‘time vs. time of observation' were noticed (*P* < 0.01) for both responses, with treatments influencing the drinking frequency at 1:00 and 4:00 pm, while time spent drinking was affected by the treatments at 9:00 am and 4:00 pm. All supplemented treatments increased (*P* < 0.01) markedly both drinking frequency (around 6 times more) and time spent drinking (around 5 times more) at 4:00 pm compared to the control group.

The β-mannanase and probiotic supplementations were able to increase (*P* < 0.01) the frequency of exploratory behavior by 65 and 51%, respectively. The same was noticed (*P* < 0.01) for the time spent in this behavior, which was increased by 61% by β-mannanase and by 63% by probiotics. Interaction ‘time vs. time of observation' was noticed for both responses (*P* < 0.05), which were not influenced by treatments at 4:00 pm.

### Effect of feeding additives on the other behaviors

Birds supplemented with combined additives increased the time wing-flapping (*P* < 0.05; Table 4), while the results observed in treatments with only β-mannanase or only probiotics were similar to control. All supplemented treatments were able to decrease (*P* < 0.01) time spent pecking in both aggressive and non-aggressive forms. The β-mannanase decreased the time in aggressive and non-aggressive pecking by 73 and 94% compared to the control group, respectively. In the same comparison, probiotics reduced the time in aggressive and non-aggressive pecking by 96 and 73%, while combined additives reduced them by 73 and 84%, respectively.

**Table 4:** Time expended (minutes/bird)^1^ in other behaviors by laying hens fed beta-mannanase and/or probiotics^2^.

**Traits**	**Treatments**				***Avg time***	***P*** **-value** ^**3**^		
	**Control**	**βM**	**PB**	**βM+PB**		**Treat**	**Obs**	**T × O**
**Scratching**								
9 h^4^	0.12	0.17	0.09	0.07	0.11	0.197	0.298	0.359
13 h^4^	0.02	0.17	0.00	0.11	0.07	SE^5^=0.03
16 h^4^	0.00	0.17	0.12	0.00	0.05			
*Avg treat*	0.05	0.15	0.07	0.06				
**Wing-flapping**								
9 h	0.02^B^	0.03^AB^	0.00^B^	0.09^A^	0.04	0.011	0.463	0.938
13 h	0.00^b^	0.05^ab^	0.00^b^	0.07^a^	0.03	SE=0.02
16 h	0.00	0.002	0.00	0.04	0.01			
*Avg treat*	0.007^B^	0.03^AB^	0.00^B^	0.07^A^				
**Leg-stretching**								
9 h	0.00	0.02	0.00	0.00	0.005	0.113	0.609	0.808
13 h	0.00	0.00	0.00	0.00	0.00	SE=0.005
16 h	0.00	0.02	0.00	0.00	0.005			
*Avg treat*	0.00	0.001	0.00	0.00				
**Wing stretching**								
9 h	0.00	0.04	0.00	0.00	0.01	0.106	0.137	0.158
13 h	0.00	0.00	0.00	0.00	0.00	SE=0.007
16 h	0.00	0.00	0.00	0.00	0.00			
*Avg treat*	0.00	0.01	0.00	0.00				
**Non-aggressive peck**								
9 h	0.31^A^	0.00^B^	0.14^B^	0.00^B^	0.11	0.005	0.259	0.331
13 h	0.12^a^	0.05^b^	0.00^b^	0.09^b^	0.06	SE=0.05
16 h	0.14^a^	0.00^b^	0.00^b^	0.00^b^	0.03			
*Avg treat*	0.19^A^	0.01^B^	0.05^B^	0.03^B^				
**Aggressive peck**								
9 h	0.57^A^	0.14^B^	0.05^B^	0.15^B^	0.23	0.002	0.103	0.588
13 h	0.24^A^	0.04^B^	0.00^B^	0.13^B^	0.10	SE=0.06
16 h	0.19^a^	0.09^b^	0.00^b^	0.00^b^	0.07			
*Avg treat*	0.33^A^	0.09^B^	0.01^B^	0.09^B^				

^1^Times that each bird performed the behavior during the observation window (15 min).

^2^ Means followed by different uppercase letters differ statistically at 5%, while lowercase letters were used to indicate differences at 10%. Comparisons were performed among treatments – lines^A, B, C, D^ within each observation time and also for averages obtained when the three observation times were polled together (indicated as ‘Avg treat'). The averages obtained when the four treatments were polled together in each observation time are also presented (indicated as ‘Avg time') and compared within the column^X, Y, Z^.

^3^Probability of treatment effect (treat), time of observation (obs), and interaction (T × O).

^4^9 h refers to the observation period from 9 AM to 9:15 AM, 13 h refers to the observation period from 13 PM to 13:15 PM, while 16 h refers to the observation period from 16 PM to 16:15 PM.

^5^Standard error.

No differences among treatments were observed in the time spent scratching, scratching wings, and scratching legs. In addition, the interaction ‘time vs. time of observation' was not significant for any response presented in this section.

### Effect of feeding additives on the frequency and score lesions

All supplemented groups showed a tendency to present fewer birds with neck injuries than the control group (*P* < 0.10; Table 5). In addition, the lesion score in the neck was also reduced (*P* < 0.05; Table 6) by all supplemented treatments. Frequency and lesion scores of neck injuries were reduced in birds fed diets containing β-mannanase by 39 and 38%, probiotics by 30 and 40%, and combined additives by 39 and 38%, respectively. However, no significant differences were observed among treatments for the frequency of lesions or for the lesion scores on the tail, cloaca, and crest.

**Table 5:** Frequency (%) of birds with lesions (disregarding the score) observed in groups of laying hens fed β-mannanase and/or probiotics1.

**Traits**	**Treatments**				**SE^2^**	***P*-value^3^**
	**Control**	**βM**	**PB**	**βM+PB**		
Tail	4	8	8	4	5	0.578
Cloaca	4	12	12	20	4	0.417
Neck	92^a^	64^b^	56^b^	56^b^	3	0.057
Comb	76	88	92	80	6	0.428

^1^Means (LSmeans) followed by different lowercase letters differ statistically at 10%.

^2^Standard error.

^3^Probability of treatment effect.

**Table 6:** Lesion score observed in laying hens fed β-mannanase and/or probiotics1.

**Traits**	**Treatments**				**SE^2^**	***P*-value^3^**
	**Control**	**βM**	**PB**	**βM+PB**		
Tail	0.04	0.16	0.08	0.12	0.09	0.952
Cloaca	0.04	0.12	0.36	0.32	0.11	0.282
Neck	1.48^A^	0.92^B^	0.92^B^	0.88^B^	0.16	0.039
Comb	1.04	0.96	1.24	1.12	0.14	0.556

^1^Means (LSmeans) followed by different lowercase letters differ statistically at 10%.

^2^Standard error.

^3^Probability of treatment effect.

## Discussion

A factor that should be taken into account when analyzing the behavior of birds is the time when they occur. Birds respond to luminous stimuli, in which the energy contained in the photons present in light is transformed into nerve stimuli that regulate the circadian rhythm, that is, the physiological responses of the animal are controlled by light. Maximum light sensitivity occurs between 10:00 am and 3:00 pm (22).

The birds were fed *ad libitum* in this trial. Even so, the frequency of eating was higher at 1:00 pm, which agrees with the findings of Rodrigues et al. (23), who observed a higher frequency of this behavior between 1:00 pm and 2:00 pm in birds raised under thermal comfort. However, (24) Giraldo et al. (24) observed a higher frequency of eating behavior between 9:00 am and 10:00 am.

Oviposition is negatively related to feed intake behavior. Consumption decreases an hour or two before oviposition but increases soon after (25). This fact may also explain the exploratory behavior observed in the birds in this study. Exploratory behavior on caged birds can be explained as dissatisfaction, as it occurs before oviposition when the bird looks for a nest (26, 27). The birds fed with the additives had a higher egg production than the control group, which may explain the increase in exploratory behavior, and, as the birds do not have access to the nest, this behavior was exacerbated by higher food intake and consequently higher posture.

In fact, it is believed that the positive effect of probiotics on egg production is due to better absorption of nutrients (28), intestinal health promotion, improved immune function, and reduced stress in birds (29). Concerning β-mannanase, by preventing the immune response that would be induced by β-mannans, this additive directs energy and nutrients to the bird's performance (30). In this study, feed consumption could not be quantified because the trial was conducted on a commercial farm. Despite the lack of this response, the most challenging environment (compared to research facilities) brings more reliable results (more applicable to production systems) in the variables of behavior and animal welfare.

Social position is another factor considered important when analyzing the feeding frequency of birds (31). Dominant birds tend to be more aggressive in feeders. In this study, the treatments were able to decrease the frequency of pecking, which may be another indication that the feed additives were able to reduce stress.

The bird hierarchy is based on the pecking of another individual of the same group, in which the hen's social position is determined by the number of individuals pecked (32). Bird pecking can be differentiated into non-aggressive and aggressive. Non-aggressive pecking is gentle and generally does not bother the receiving bird, unlike aggressive pecking which consists of plucking the feather from its receiver. Both patterns are defined as abnormal behaviors (33). Feather pecking may also be associated with negative affective states such as fear (34). Cheon et al. (35) observed that pecking peaks occurred close to feeding time. No effect of time was observed in the present study, but the feed additives were able to decrease the pecking behavior, which indicates that the animals were less stressed.

Feather pecking is a stress-induced neuropsychological disorder in birds. Intestinal dysbiosis and inflammation are common features of these disorders. Thus, this behavior may be linked to a set of consequences of dysregulated communication between the gut and the brain (36, 37). Mindus et al. (36) demonstrated that the use of probiotics had an immunological effect by increasing spleen T cells and cecal tonsils, in addition to limiting the dysbiosis of the cecal microbiota. Thus, with the results obtained in this study, it is possible to state that probiotics, by regulating the intestinal microbiota, were able to reduce stress. On the other hand, β-mannanase was able to improve the welfare of the animals by decreasing intestinal and systemic inflammation caused by β-mannans and beneficially modulating the microbiota.

It is also worth mentioning the lesions on the crest when discussing the dominance effects. These lesions are also associated with dominance and aggression among birds, mainly due to crest pecking and neck aggression (33, 38). In this study, the control group had a greater frequency of neck injuries, in addition to having a higher number of pecks, as already shown, which may be an indication that the treatments reduced stress in the birds, by reducing fights.

It is also important to consider that drinking behavior is related to eating behavior. As already stated, birds normally have two peak moments of these behaviors, which are 2–3 h after the light is turned on and 2–3 h before the light is turned off (39). In the present study, the birds showed a higher frequency of this behavior at 4:00 pm, which agrees with other authors (23, 24).

Another important factor to be taken into consideration is the lack of environmental enrichment in the facilities used for the project. In caged birds, the lack of attractants can be a stimulus for greater drinking behavior, as it is performed as a distraction rather than thirst (40). However, in this study, all animals remained under the same conditions and showed a higher frequency of drinking behavior with the use of treatments, which indicates that there was an influence on the animals, which led them to drink and eat more.

Regarding standing and sitting behavior, is important to add that as the hens get older, they sit more and stand less (41). In this study, hens supplemented with additives performed standing behavior more frequently and for a longer time, which could indicate greater exercise and is beneficial for leg muscles and bones, especially in caged birds (42). Mohammed et al. (43) observed that broilers supplemented with probiotics spent more time standing in the latency-to-lie test. They also observed higher tibial physical parameters (length, weight, and strength), in addition to higher concentrations of calcium and phosphorus in the blood. The supplemented hens also performed sitting behavior for less time and less frequently. Sitting behavior is linked to nesting behavior since it precedes oviposition (44). Struelens et al. (45) showed that hens spent more time sitting and less time standing in the nest with flaps, which indicates a stable pre-laying behavior, that is seen as something positive. In this research, the behaviors were not differentiated during oviposition time, which makes it difficult to interpret.

Concerning walking behavior, De Hass et al. (46) observed that the mean duration of walking in laying hens at 6 weeks of age was inversely proportional to the plasma corticosterone level. Thus, the higher the average walking distance of the animals, the lower the stress and the greater the welfare. Lei et al. (47) and Sohail et al. (4) also showed lower levels of corticosterone in animals supplemented with probiotics. In this study, probiotic treatment was similar to control, but an interaction between treatment and time was shown. In other periods, β-mannanase and combined additives decreased this behavior. Thus, more studies should be carried out to understand further this relationship.

Regarding wing-flapping behavior, Zimmerman et al. (48) conducted a study in which birds experienced three events, positive, negative and neutral. In positive events, the birds presented the flapping of wings, which was then recognized as a comfort movement. Thus, we believe that in this study, the birds fed with both additives, by demonstrating this behavior more than the control group, had a beneficial effect on their welfare, due to the probable modulation of the microbiota.

## Conclusion

The present study indicates that a multi-strain probiotic additive, β-mannanase, and their association were able to perform alterations in the behavior of lightweight laying hens (36 weeks old). β-mannanase and probiotics increased the frequency and the time that laying hens spent performing feeding, drinking and exploring when compared to the control treatment. Both additives also promoted a higher frequency in standing behavior and decreased the time spent on sitting behaviors. Combined additives showed less frequency and time in sitting behaviors and increased wing-flapping behavior. In addition, all the treatments were able to reduce pecking and neck lesions. Thus, adding β-mannanase and probiotics to laying hen diets reared on cage systems is an effective strategy to improve their welfare. Future studies are needed to further elucidate the connection between those additives and the welfare biomarkers.

## Data availability statement

The raw data supporting the conclusions of this article will be made available by the authors, without undue reservation.

## Ethics statement

The animal study was reviewed and approved by Institutional Ethics Committee on the Use of Animals (CEUA/UFRGS) under protocol number 39783.

## Author contributions

CC: conceptualization, methodology, data acquisition, formal analysis, investigation, writing original draft preparation, and writing the final paper. IA: supervision, data curation, conceptualization, methodology, writing original draft preparation, and review. GG: data acquisition, visualization, and review. GM, NC, and TS: data acquisition. RM: supervision and review. MS: conceptualization, funding acquisition, and review. All authors contributed to the article and approved the submitted version.

## Conflict of interest

Author MS was employed by Elanco Animal Health, São Paulo, Brazil. The remaining authors declare that the research was conducted in the absence of any commercial or financial relationships that could be construed as a potential conflict of interest.

## Publisher's note

All claims expressed in this article are solely those of the authors and do not necessarily represent those of their affiliated organizations, or those of the publisher, the editors and the reviewers. Any product that may be evaluated in this article, or claim that may be made by its manufacturer, is not guaranteed or endorsed by the publisher.
